# Novel p22 and p30 dual-proteins combination based indirect ELISA for detecting antibodies against African swine fever virus

**DOI:** 10.3389/fvets.2023.1093440

**Published:** 2023-02-10

**Authors:** Jianda Li, Jian Jiao, Na Liu, Sufang Ren, Hao Zeng, Jun Peng, Yuyu Zhang, Lihui Guo, Fei Liu, Tingting Lv, Zhi Chen, Wenbo Sun, Nataliia Hrabchenko, Jiang Yu, Jiaqiang Wu

**Affiliations:** ^1^Shandong Key Laboratory of Animal Disease Control and Breeding, Institute of Animal Science and Veterinary Medicine, Shandong Academy of Agricultural Sciences, Jinan, China; ^2^School of Veterinary Medicine, Qingdao Agricultural University, Qingdao, China; ^3^School of Life Sciences, Shandong Normal University, Jinan, China; ^4^College of Animal Science and Technology, Shandong Agricultural University, Tai'an, China

**Keywords:** African swine fever virus, indirect ELISA, p22, p30, serological diagnosis

## Abstract

**Introduction:**

African swine fever virus (ASFV) infection is one of the most complex and fatal hemorrhagic viral diseases, causing a devastating loss to the swine industry. Since no effective vaccine is available, prevention and control of ASFV heavily depends on early diagnostic detection.

**Methods:**

In this study, a novel indirect ELISA was established for detecting antibodies against ASFV using dual-proteins, p22 and p30. Recombinants p22 and p30 were expressed and purified from *E.coli* vector system by recombined plasmids pET-KP177R and pET-CP204L. p22 and p30 were mixed as antigens for developing the indirect ELISA.

**Results:**

Through optimizing coating concentrations of p30 and p22, coating ratio (p30: p22 = 1:3), and serum dilution (as 1:600), the established ELISA performed higher specificity, sensitivity, and repeatability against ASFV-positive serum. Furthermore, 184 clinical serum samples from suspected diseased pigs were verified the established ELISA in clinical diagnosis. The results showed that compared with two commercial ELISA kits, the established ELISA possessed higher sensitivity and almost uniform coincidence rate.

**Conclusion:**

The novel indirect ELISA based on dual-proteins p30 and p22 performed a valuable role in diagnostic detection of ASFV, providing a broad insight into serological diagnostic methods of ASFV.

## 1. Introduction

African swine fever (ASF) is an acute and highly contact infectious disease, caused by African swine fever virus (ASFV). ASFV infection induced high fever, lethargy, and death in pigs, causing a devastating loss to the swine industry. ASF was firstly found in Kenya in 1909 and reported in 1921 (1). Subsequently, ASFV spread to Central and Eastern Europe (2). In China, ASFV infection first occurred in Liaoning Province in August 2018, and subsequently spread to all provinces of China (3). Moreover, genotype I ASFVs and low virulent genotype II ASFVs occurred in China (4, 5). Since no commercial vaccine is available, the emergence of ASFVs presents new challenges for the early diagnosis and control of ASF.

As the only member of the family *Asfarviridae*, ASFV is an enveloped virus containing 170–190 kb double-stranded DNA. The ASFV genome includes more than 150 open reading frames, encoding 54 structural proteins and more than 100 nonstructural proteins (6). Among these proteins, p22, encoded by KP177R gene, is a structural protein located at the inner envelope of ASFV virion (7). Recently, a function genomics has shown that p22 protein interacts with host proteins involved in several cellular function, including cell signaling transduction, cell structure, and virus binding (8). Although a recombinant ASFV lacking p22 has no effect on pathogenicity and virulence of ASFV, immunization with p22 could induce a higher antibody titer, indicating that p22 has potential as a target for serological diagnosis (9, 10). p30 protein is one of the most immunogenic structural proteins in the ASFV virion, which is encoded by CP204L gene (11). During ASFV infection of macrophage, the expression of p30 is detected at 2–4 h post-infection and then persists throughout the infection cycle (12). Thus, p30 is considered to be an ideal diagnostic protein using for diagnosis of ASFV in the early state of infection.

Since no commercial vaccines against this disease currently, early detection and diagnosis play a vital role in the prevention and control of ASFV. In addition to molecular diagnostic method, serological detection is another method for virus infection, which is conducive to identify infected animals and eradicate the potential risk (13, 14). Although molecular diagnostic methods are very important for the early diagnosis and prevention of ASF, the characteristics of low cost and convenience of serological methods are more suitable for large-scale field epidemiological investigation (14–16). The establishment of reliable serological diagnostic methods is closely related to the antigenicity of the selected antigens (17, 18). Enzyme-linked immunosorbent assay (ELISA) is a designated experiment specified by OIE (World Organization for Animal Health) for international trade to detect specific antibodies to ASFV. Screening several viral proteins with higher reactivity is very important for establishing reliable serological diagnostic methods and avoiding unnecessary biosafety problems (19, 20). In this study, we expressed and purified ASFV p22 and p30 proteins, and established an indirect ELISA method for detecting antibodies against ASFV.

## 2. Materials and methods

### 2.1. Serum samples

ASFV-positive serum was purchased from China Institute of Veterinary Drug Control. All clinical swine sera were donated from Vland Biotech (China). The negative sera against ASFV, and the positive sera against porcine circovirus type 2 (PCV2), porcine pseudorabies virus (PRV), classical swine fever virus (CSFV), porcine reproductive and respiratory syndrome virus (PRRSV), and *Haemophilus parasuis* (HPS) were stored in our lab.

### 2.2. Sequence analysis and optimization

The amino acid sequences of p22 and p30 were analyzed for immunogenicity, hydrophilicity and transmembrane region by IEDB database (http://tools.immuneepitope.org/bcell/). According to *Escherichia coli* (*E.coli*) expression systems, the sequences of KP177R gene and CP204L gene were optimized and synthesized based on ASFV HLJ/18 strain (Accession number MK333180.1). Subsequently, the synthesized sequences were cloned into pEASY-Blunt vector by gene synthesis corporation.

### 2.3. Expression of p22 and p30

To construct the expression plasmids of p22 and p30, the sequences of CP204L and KP177R were amplified by PCR using the primers containing *BamHI* and *XhoI* restriction enzyme sites (Table 1). After verification by sequencing, the sequences of CP204L and KP177R were inserted into pET-32a vector. The plasmids recombinants pET-KP177R and pET-CP204L were transformed into *E.coli* BL21(DE3) cells. The recombinants *E.coli* were cultured in LB medium and the condition of proteins expression were optimized, such as culture time, temperature, IPTG concentration. The immunogenicity evaluation was performed with standard ASFV-positive serum (China Institute for Veterinary Drug Control) and His-Tag monoclonal antibody (Proteintech).

**Table 1:** The sequences of primers.

**Primers name**	**Primers sequences**
CP204L-R	*GGATCC*ATGGATTTCATCCTGAATATC
CP204L-F	*CTCGAG*TTTTTTTTTCAGCAGTTTAA
KP177R-R	*GGATCC*AAAAAACAGCAGCCGCCGA
KP177R-F	*CTCGAG*TTATGCGTGTTTATGATTAC

### 2.4. Purification of p22 and p30

After optimizing the culture conditions, the *E.coli* were centrifuged to get the pellets (4,000 rpm, 30 min, 4°C) and then resuspended in pre-cold PBS on ice for ultrasonication. According the manufacturer's protocol, the supernatants were collected and filtered through a 0.22 μm filter and purified using a Ni-NTA resin-based column (GE Healthcare) following centrifuging at 12,000 rpm for 30 min. After eluting with elution buffer, the fractions were dissolved in PBS containing 5% glycerol and concentrated by ultrafiltration. The protein concentration was determined by a BCA Protein Assay Kit (Thermo Fisher). The purified p22 and p30 proteins were verified using sodium dodecyl sulfate-polyacrylamide gel electrophoresis (SDS-PAGE) and Coomassie blue staining.

### 2.5. Western blot

Following separation by SDS-PAGE, proteins were transferred onto PVDF membrane. After blocking with 5% skim milk for 2 h, the membrane was incubated with standard ASFV-positive serum (1:1,000) overnight at 4°C. Then, the membrane was incubated with HRP-conjugated goat anti-pig secondary antibodies (1:8,000, Abcam). Finally, the membrane was visualized in Bioanalytical imaging system.

### 2.6. Establishment of indirect ELISA

#### 2.6.1. Determination of coating concentration and serum concentration

The coating concentration and serum concentration were optimized by checkerboard titration (21). Briefly, p22 and p30 were diluted (1:20–1:400) and coated on 96-well microtitration plates. ASFV-positive and ASFV-negative sera with different dilutions were incubated. After incubating with HRP-conjugated goat anti-swine IgG (H+L) antibody and stopping with stop solution, the plates were quantified using a microplate reader at 450 nm. Coating concentration and serum concentration were developed the best reaction condition by determining the negative sample (N) value, positive sample (P) value, and P/N ratio. Based this condition, the optimal dilution of horseradish peroxidase-conjugated secondary antibodies was further determined.

#### 2.6.2. Determination of cut-off value, specificity, sensitivity, and repeatability

To determine the cutoff vale of the established ELISA, 50 ASFV-negative serum samples were evaluated. The competitive ELISA based on p32 (produced by ID.vet) was used as a reference. The mean value (X) and standard deviation (SD) of 50 samples were calculated. Negative ≤ X + 2 × SD. Positive ≥ X + 3 × SD. The middle is considered as the suspicious range.

For verifying the specificity of the established indirect ELISA, pig serums positive against other pig pathogens were tested, including pseudorabies virus (PRV), porcine reproductive and respiratory syndrome virus (PRRSV), porcine circovirus 2 (PCV2), classical swine fever virus (CSFV), *Haemophilus parasuis* (HPS). The ASFV-positive serum and the serum from specific pathogen-free (SPF) pig was used as positive and negative control, respectively.

According to the optimized condition, the sensitivity was carried out by testing the serial dilution multiple of ASFV-positive serum (1:200–1:15,000).

To assess the repeatability of the indirect ELISA, ASFV-positive blood samples (determined by ID.vet competitive ELISA) were selected for intra-assay and inter-assay repeatability experiments. For inter-assay variability, each sample was retested three times on plates of different batches. For intra-assay variability, each sample was repeated 3 times on the same plate at the same time. The results are expressed as the coefficient of variation (CV), that is, the ratio of the SD of each group of samples to the average OD_450_ value.

### 2.7. Detection of clinical samples

A total of 184 serum samples from suspected diseased pigs was blinded by the established indirect ELISA and two commercial ELISA kits (ID.vet and JUNO). All the serum samples were detected by p22 and p30 dual-proteins combination based indirect ELISA method in this study. The coincidence was calculated.

## 3. Results

### 3.1. Expression and purification of p22 and p30

**Figure 1:**
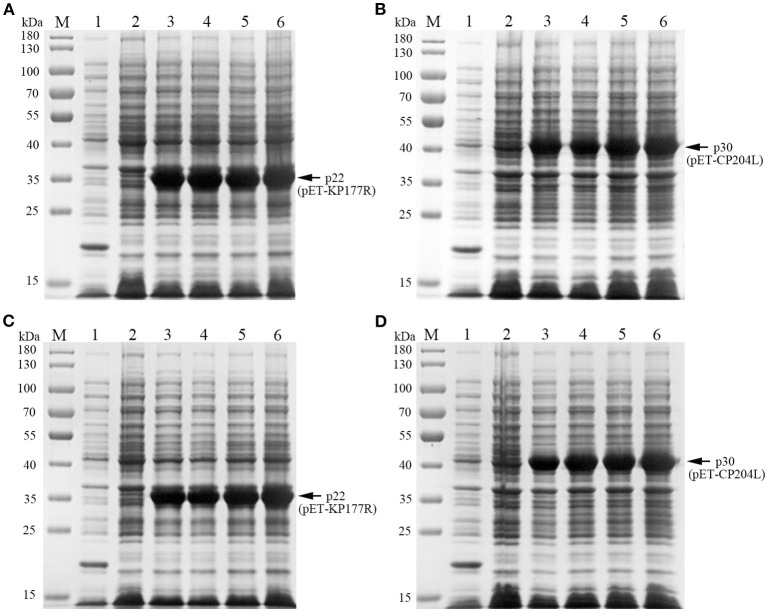
Exploration of p22 and p30 proteins induction conditions. **(A, B)** Determination of optimal IPTG concentration of p22 **(A)** and p30 **(B)**. M, Marker; (1) pET-32a empty carrier; (2) Before induction; (3–6) IPTG concentration at 0.1, 0.4, 0.7, and 1.0 mM. **(C, D)** Determination of the best OD_600_ of p22 **(C)** and p30 **(D)**. M, Marker; (1) pET-32a empty carrier; (2) Before induction; (3–6) OD_600_ at 0.4, 0.6, 0.8, 1.

**Figure 2:**
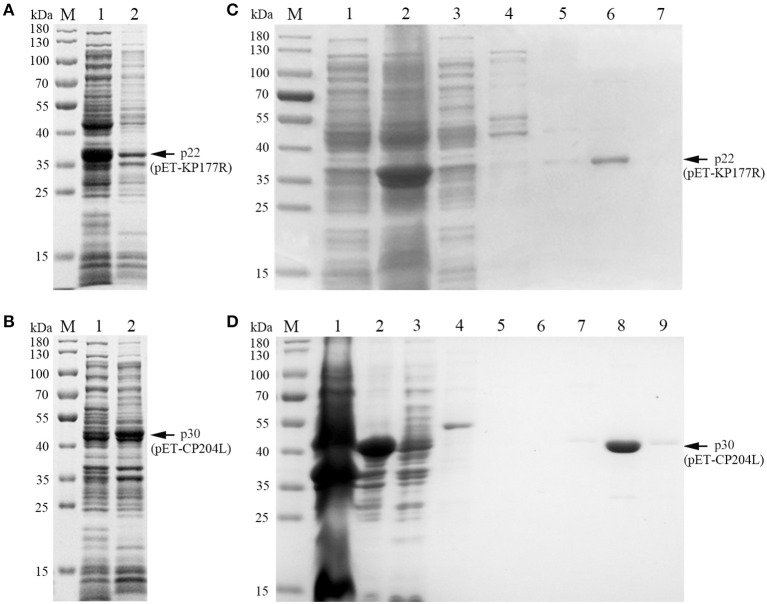
Soluble analysis and purification of p22 and p30 proteins. **(A, B)** Soluble analysis of p22 protein **(A)** and p30 protein **(B)**. M, Marker; (1) Supernatant after ultrasound; (2) Precipitation after ultrasound. **(C, D)** The purification results of p22 protein **(C)** and p30 protein **(D)**. **(C)** M, Marker; (1–7) Flow through fluid, supernatant after ultrasound, 20, 40, 50, 100, and 200 mM imidazole. **(D)** M, Marker; (1–9) Precipitation after ultrasound, supernatant after ultrasound, flow through fluid, 20, 40, 50, 100, 200, and 200 mM imidazole.

To explore the optimal conditions for the expression of p22 and p30, the recombinants *E.coli* (containing pET-KP177R or pET-CP204L) was cultivated with different concentrations of IPTG for 4 h at 37°C. The results showed induction expression using 0.1–1.0 mM IPTG had no effect on the expression of p22 and p30 (Figures 1A, B). When the OD_600_ value reach 0.8–1.0, the recombinants *E.coli* were more conducive to induction expression (Figures 1C, D). We further found that p22 was mainly expressed in the supernatant, while p30 was expressed both in the supernatant and precipitation (Figures 2A, B). The soluble protein fraction was purified with Ni-NTA Sepharose, and the result showed that 100 mM imidazole was more conducive to elution of p22 protein and 200 mM imidazole was beneficial to elute p30 protein (Figures 2C, D).

### 3.2. Immunogenicity of recombinant protein p22 and p30

**Figure 3:**
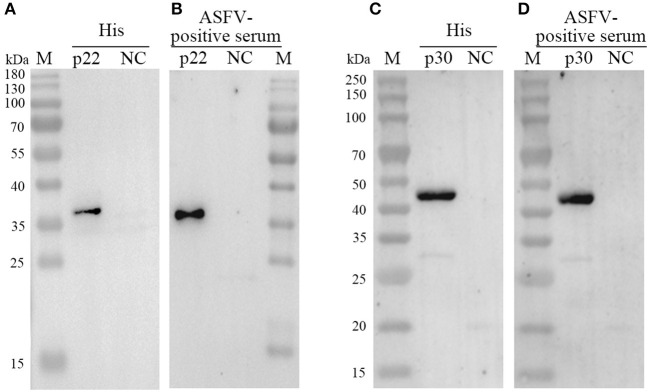
Determination the expression of p22 and p30. **(A, C)** The expression of p22 protein **(A)** and p30 protein **(C)** was determined by anti-His antibody. **(B, D)** The expression of p22 protein **(B)** and p30 protein **(D)** was immunoreactive with ASFV-positive serum.

Both of purified p22 and p30 were primarily verified by Western blot and performed a strong immunoreactivity with anti-His antibody (Figures 3A, C). Furthermore, the results of Western blot showed that the purified p22 and p30 protein specifically reacted with ASFV-positive serum (Figures 3B, D). Taken together, the purified p22 and p30 exhibited higher immunogenicity.

### 3.3. Optimization of experimental conditions for ELISA

**Figure 4:**
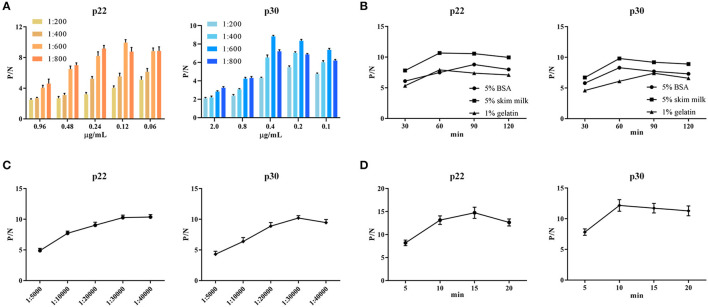
Optimal conditions of ELISA. **(A)** Optimal concentration of coating protein and serum dilution. **(B)** Optimal blocking solution and best blocking time. **(C)** Determination of the optimum dilution of enzyme-labeled secondary antibody. **(D)** Optimum substrate-enzyme interaction time.

To determine the optimal conditions, the checkerboard titrations were performed. The results showed that the optimum coated concentration of p22 and p30 was determined at 0.12 and 0.4 μg/mL, and the optimum dilution ratio of serum as primary antibody was 1:600 (Figure 4A). For blocking conditions, we found compared with 5% BSA and 1% gelatin, using 5% skim milk for 60 min exhibited higher performed a higher blocking effect (Figure 4B). Moreover, the dilution ratio of secondary antibody and the reaction time of substrate-enzyme were explored. The result showed for p22, the optimum dilution of secondary antibody reached 1:40,000 (Figure 4C) and the optimum reaction time is 15 min (Figure 4D); for p30, the optimum dilution of secondary antibody was 1:30,000 and the reaction time of substrate-enzyme is 10 min (Figures 4C, D). To develop the indirect ELISA based p22 and p30, the coating ratio of both proteins were evaluated. Based on calculating P/N value, we found that the optimum volume ratio of p30 to p22 reached 1:3 (Table 2).

**Table 2:** Coating volume ratio of p30 and p22.

**Volume ratio of p30 to p22**	**p30**	**p22**	**1:1**	**2:1**	**1:2**	**3:1**	**1:3**
ASFV-positive sera	2.920	1.394	1.537	1.672	1.261	1.502	0.835
ASFV-negative sera	0.474	0.195	0.244	0.219	0.164	0.186	0.101
P/N	6.2	7.2	6.3	7.6	7.7	8.1	8.3

### 3.4. Determination of cut-off value, sensitivity, repeatability, and specificity

**Figure 5:**
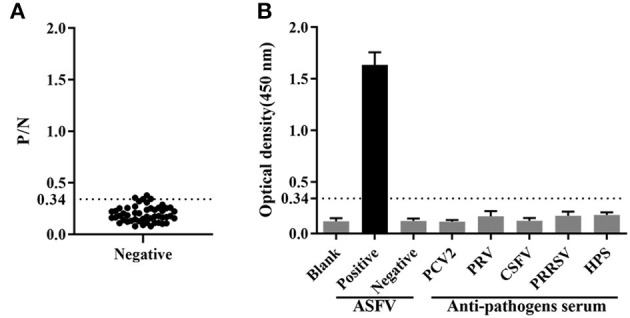
Determination of sensitivity and specificity. **(A)** The cut-off value of the established ELISA. **(B)** The specificity was determined using positive serums against PCV2, PRV, CSFV, PRRSV and HPS.

Fifty ASFV-negative serum samples (determined by ID.vet competitive ELISA) were used to determine the cut-off value of the established ELISA. As shown in Figure 5A, the mean value of ASFV-negative serum was 0.174, and the cut-off value was determined to be 0.34. For assessing the sensitivity of this ELISA, ASFV-positive serums were diluted to detect. The results showed compared with ID.vet ELISA kit, the established ELISA performed higher sensitivity (Table 3). To determine the repeatability of this ELISA, 4 selected ASFV-positive serums were performed by intra-assay and inter-assay. We found the intra-assay coefficients of variation (CV) ranged from 2.0 to 4.5% And the inter-assay CV ranged from 2.5 to 5.5% (Table 4), indicating that the indirect ELISA exhibited higher repeatability. To assess the specificity of this ELISA, the positive serums against PCV2, PRV, PRRSV, CSFV, and HPS were detected. The results showed that all these serums were negative (Figure 5B), indicating the established ELISA possessed high specificity.

**Table 3:** Determination of sensitivity.

**Test kit**	**Dilution ratios**	**1:200**	**1:400**	**1:800**	**1:1,600**	**1:3,200**	**1:6,400**	**1:12,800**	**1:25,600**
p22 and p30	OD_450_	2.387	2.105	1.791	1.379	1.086	0.732	0.435	0.216
	P/N	+	+	+	+	+	+	+	-
ID.vet	OD_450_	0.172	0.285	0.397	0.511	0.654	/	/	/
	P/N	+	+	+	+	-	-	-	-

**Table 4:** Determination of repeatability by inter-assay and intra-assay.

**Sample no**.	**Intra-assay**		**Inter-assay**	
	**Results**	**CV%**	**Results**	**CV%**
1	2.513 ± 0.072	2.86	2.525 ± 0.135	5.34
2	1.404 ± 0.045	3.21	1.372 ± 0.025	2.55
3	1.64 ± 0.033	2.01	1.684 ± 0.054	3.21
4	0.835 ± 0.034	4.07	0.821 ± 0.39	4.75

### 3.5. Clinical samples detection

Total 184 pig blood samples were detected by the above established indirect ELISA, and 99 positive samples and 83 negative samples were detected by ID.vet competitive ELISA kit. Among 184 samples, the results of 174 samples detected by the established ELISA were consistent with that of ID.vet ELISA, the coincidence rate of the established ELISA arrived at 94.6% (compared with ID.vet ELISA) (Table 5). Moreover, the sensitivity of the established ELISA was higher than that of other ELISA kits (Table 5). Several negative samples determined by ID.vet ELISA were identified as positive samples and suspicious samples by the established ELISA. Taken together, the indirect ELISA based on p22 and p30 could be adapted to clinical serological diagnosis.

**Table 5:** Coincidence rate of clinical samples.

**ELISA kit**	**Sample numbers**	**Positive samples**		**Negative samples**		**Suspicious samples**	**Samples with different results**	**Coincidence rate (Compared with ID.vet)**
		**Numbers**	**Positive detection rate**	**Numbers**	**Negative detection rate**			
p22 and p30	184	101	54.9%	79	42.9%	4	10	94.6%
ID.vet	184	99	53.8%	83	45.1%	2	/	/
JUNO	184	96	52.2%	87	47.3%	1	7	96.2%

## 4. Discussion

ASF is a global epidemic disease with high mortality, causing a serious impact on the global swine industry. Considering that there is no effective vaccine to prevent and control ASFV, the only effective measure is to diagnostic analysis and eliminate infected animals. Thus, highly sensitive and specific diagnostic analysis performed an important role in rapid detection of ASFV. Due to the advantages of low cost, high sensitivity, and strong specificity, ELSIA is recommended as the primary method for detecting ASFV antibody (22).

ASFV encodes more than 50 structural proteins. It is necessary to develop ELISA based on the viral proteins expressed in different stages of viral infection. At present, several commercial ELISA kits were effective and available for detecting ASFV antibodies. For example, the multi-antigen indirect ELISA kit based on the mixture of three recombinant proteins p32, p62 and p72 produced by ID.vet in France. Besides, several studies have used p30, p54, p72, and other viral proteins as coating proteins for establishing ELISA to detect ASFV antibodies. p72 is a late structural protein of ASFV, which is located in the middle or surface layer of viral particles (23). ASFV p72 gene possesses highly conserved sequence, inducing a strong immune response (24). Moreover, a recent study has used p72 protein expressed by eukaryotic system as coating antigen to establish an blocking ELISA (25). p54 protein is an early structural protein in ASFV infection, which involves in viral replication, transfection, and maintenance of structural stability (23). An indirect ELISA detection method based on the p54 protein produced by baculovirus expression system was developed and performed higher coincidence rate compared with the commercial kits (26). pp62 is an important structural protein of ASFV, cleaved into p35, p15 and p8 proteins by s273r protease during maturation of viral particle (7). The recombinant pp62 protein using baculovirus expression system has been used as coating protein for establishing ELISA, which is recommended by OIE resulting its sensitivity and specificity (27). CD2v is a membrane protein embedded in the outer surface of the virus capsule and a late expression protein of ASFV (28), which can lead to the adsorption of erythrocytes on the surface of virus infected cells and contribute to their diffusion in the host (29). ASFV CD2v protein was expressed in CHO-K1 cells and established an indirect ELISA method with good specificity and sensitivity (21).

Since ELISA based on different ASFV proteins has different characteristics, it is necessary to continuously explore other viral proteins of ASFV that can be used for specific antibody detection, and select different antigen combinations to further improve ELISA detection methods. p22 is an early transcribed, structural protein localized at the inner envelope of ASFV particle. Although recent study has confirmed p22 protein did not seem to be involved in viral replication or virulence in pigs by developing a recombinant ASFV lacking the KP177R gene, p22 protein could interact with cellular proteins to participate in viral binding, signal transduction, and cell adhesion (8). Recently, a blocking ELISA based on p22-monoclonal antibody showed higher s sensitivity and specificity for detecting ASFV antibodies (30). p30, a membrane phosphorylated protein, is expressed in the early stage of ASFV infection and plays a significant role in virus internalization (31, 32). Recent research has showed p30 could interact with 7 cellular proteins to involve in viral internalization mediated by clathrin and micropinocytosis, and might regulate innate immunity by interacting with innate immune regulators (33). Furthermore, an indirect ELISA based on p30 expressed by prokaryotic expression system has been established and showed higher specificity (34). In this study, based on the recombinant proteins p22 and p30 were expressed in prokaryotic expression system, an indirect ELISA was developed and showed higher sensitivity and specificity.

## 5. Conclusion

An indirect ELISA based on p30 and p22 protein was established. Through detection of standard ASFV-positive serums, positive serums against other virus, and negative serums, the sensitivity and specificity of this ELISA was determined. Our study provides a broad insight into serological diagnostic methods of ASFV antibodies, but it still needs to be further verified by more pig serums from different sources to expand the experimental data and improve the detection method.

## Data availability statement

The raw data supporting the conclusions of this article will be made available by the authors, without undue reservation.

## Ethics statement

The animal study was reviewed and approved by Shandong Province Animal Ethics Committee.

## Author contributions

JL, JJ, and NL performed the experiments and wrote the manuscript. SR, HZ, LG, JP, and TL were responsible for samples collection. YZ, FL, ZC, WS, and NH corrected the manuscript. JW and JY initiated the study, designed the experiments, and supplied the manuscript. All authors reviewed the manuscript. All authors contributed to the article and approved the submitted version.

## References

[1] Blasco R Agüero M Almendral JM Viñuela E. Variable and constant regions in African swine fever virus DNA. Virology. (1989) 168:330–8. 10.1016/0042-6822(89)90273-0 2464873

[2] Costard S Wieland B de Glanville W Jori F Rowlands R Vosloo W. African swine fever: how can global spread be prevented? Philos Trans R Soc Lond B Biol Sci. (2009) 364:2683–96. 10.1098/rstb.2009.0098 19687038 PMC2865084

[3] Ge S Li J Fan X Liu F Li L Wang Q. Molecular characterization of african swine fever virus, China, 2018. Emerg Infect Dis. (2018) 24:2131–3. 10.3201/eid2411.181274 30141772 PMC6199985

[4] Sun E Huang L Zhang X Zhang J Shen D Zhang Z. Genotype I African swine fever viruses emerged in domestic pigs in China and caused chronic infection. Emerg Microbes Infect. (2021) 10:2183–93. 10.1080/22221751.2021.1999779 34709128 PMC8635679

[5] Sun E Zhang Z Wang Z He X Zhang X Wang L. Emergence and prevalence of naturally occurring lower virulent African swine fever viruses in domestic pigs in China in 2020. Sci China Life Sci. (2021) 64:752–65. 10.1007/s11427-021-1904-4 33655434

[6] Duan X Ru Y Yang W Ren J Hao R Qin X. Research progress on the proteins involved in African swine fever virus infection and replication. Front Immunol. (2022) 13:947180. 10.3389/fimmu.2022.947180 35935977 PMC9353306

[7] Alejo A Matamoros T Guerra M Andrés G. A proteomic atlas of the African swine fever virus particle. J Virol. (2018) 92:e01293-18. 10.1128/JVI.01293-18 30185597 PMC6232493

[8] Zhu X Fan B Zhou J Wang D Fan H Li B. High-throughput method to analyze the interaction proteins with p22 protein of african swine fever virus *in vitro*. Front Vet Sci. (2021) 8:719859. 10.3389/fvets.2021.719859 34552974 PMC8450437

[9] Vuono EA Ramirez-Medina E Pruitt S Rai A Espinoza N Velazquez-Salinas L. Evaluation of the function of the ASFV KP177R gene, encoding for structural protein p22, in the process of virus replication and in swine virulence. Viruses. (2021) 13:986. 10.3390/v13060986 34073222 PMC8227490

[10] Díaz C Salát J Kolarová DB Celer V Frébort I. Examination of immunogenic properties of recombinant antigens based on p22 protein from African swine fever virus. J Vet Res. (2022) 66:297–304. 10.2478/jvetres-2022-0043 36349136 PMC9597933

[11] Petrovan V Yuan F Li Y Shang P Murgia MV Misra S. Development and characterization of monoclonal antibodies against p30 protein of African swine fever virus. Virus Res. (2019) 269:197632. 10.1016/j.virusres.2019.05.010 31129172

[12] Lithgow P Takamatsu H Werling D Dixon L Chapman D. Correlation of cell surface marker expression with African swine fever virus infection. Vet Microbiol. (2014) 168:413–9. 10.1016/j.vetmic.2013.12.001 24398227 PMC3969584

[13] Arias M Jurado C Gallardo C Fernández-Pinero J Sánchez-Vizcaíno JM. Gaps in African swine fever: analysis and priorities. Transbound Emerg Dis. (2018) 65 Suppl 1:235–47. 10.1111/tbed.12695 28941208

[14] Zsak L Borca MV Risatti GR Zsak A French RA Lu Z. Preclinical diagnosis of African swine fever in contact-exposed swine by a real-time PCR assay. J Clin Microbiol. (2005) 43:112–9. 10.1128/JCM.43.1.112-119.2005 15634958 PMC540100

[15] Gallardo C Fernández-Pinero J Arias M. African swine fever (ASF) diagnosis, an essential tool in the epidemiological investigation. Virus Res. (2019) 271:197676. 10.1016/j.virusres.2019.197676 31362027

[16] Dixon LK Chapman DA Netherton CL Upton C. African swine fever virus replication and genomics. Virus Res. (2013) 173:3–14. 10.1016/j.virusres.2012.10.020 23142553

[17] Gallardo C Reis AL Kalema-Zikusoka G Malta J Soler A Blanco E. Recombinant antigen targets for serodiagnosis of African swine fever. Clin Vaccine Immunol. (2009) 16:1012–20. 10.1128/CVI.00408-08 19420186 PMC2708404

[18] Pérez-Filgueira DM González-Camacho F Gallardo C Resino-Talaván P Blanco E Gómez-Casado E. Optimization and validation of recombinant serological tests for African Swine Fever diagnosis based on detection of the p30 protein produced in Trichoplusia ni larvae. J Clin Microbiol. (2006) 44:3114–21. 10.1128/JCM.00406-06 16954235 PMC1594705

[19] Ward MP Tian K Nowotny N. African Swine Fever, the forgotten pandemic. Transbound Emerg Dis. (2021) 68:2637–9. 10.1111/tbed.14245 34499823

[20] Galindo I Alonso C. African swine fever virus: a review. Viruses. (2017) 9:103. 10.3390/v9050103 28489063 PMC5454416

[21] Jiang W Jiang D Li L Wan B Wang J Wang P. Development of an indirect ELISA for the identification of African swine fever virus wild-type strains and CD2v-deleted strains. Front Vet Sci. (2022) 9:1006895. 10.3389/fvets.2022.1006895 36157191 PMC9493115

[22] Gallardo C Nieto R Soler A Pelayo V Fernández-Pinero J Markowska-Daniel I. Assessment of African swine fever diagnostic techniques as a response to the epidemic outbreaks in eastern european union countries: How to improve surveillance and control programs. J Clin Microbiol. (2015) 53:2555–65. 10.1128/JCM.00857-15 26041901 PMC4508403

[23] Wang Y Kang W Yang W Zhang J Li D Zheng H. Structure of African swine fever virus and associated molecular mechanisms underlying infection and immunosuppression: a review. Front Immunol. (2021) 12:715582. 10.3389/fimmu.2021.715582 34552586 PMC8450572

[24] Kaneko H Iida T Aoki K Ohno S Suzutani T. Sensitive and rapid detection of herpes simplex virus and varicella-zoster virus DNA by loop-mediated isothermal amplification. J Clin Microbiol. (2005) 43:3290–6. 10.1128/JCM.43.7.3290-3296.2005 16000450 PMC1169145

[25] Caixia W Songyin Q Ying X Haoyang Y Haoxuan L Shaoqiang W. Development of a blocking ELISA Kit for Detection of ASFV antibody based on a monoclonal antibody against full-length p72. J AOAC Int. (2022) 105:1428–36. 10.1093/jaoacint/qsac050 35595230

[26] Liang Y Cao C Tao H Tang Y. Eukaryotic expression of African swine fever virus P54 protein and development of an indirect ELISA for detection of antibody against ASFV. Vet Sci China. (2014) 44:373–8. 10.16656/j.issn.1673-4696.2014.04.006

[27] Gallardo C Blanco E Rodríguez JM Carrascosa AL Sanchez-Vizcaino JM. Antigenic properties and diagnostic potential of African swine fever virus protein pp62 expressed in insect cells. J Clin Microbiol. (2006) 44:950–6. 10.1128/JCM.44.3.950-956.2006 16517882 PMC1393094

[28] Goatley LC Dixon LK. Processing and localization of the African swine fever virus CD2v transmembrane protein. J Virol. (2011) 85:3294–305. 10.1128/JVI.01994-10 21248037 PMC3067853

[29] Wang F Zhang H Hou L Yang C Wen Y. Advance of African swine fever virus in recent years. Res Vet Sci. (2021) 136:535–9. 10.1016/j.rvsc.2021.04.004 33882382

[30] Tsegay G Tesfagaber W Zhu Y He X Wang W Zhang Z. Novel P22-monoclonal antibody based blocking ELISA for the detection of African swine fever virus antibodies in serum. Biosaf Health. (2022) 4:234–43. 10.1016/j.bsheal.2022.04.00236366433

[31] Wu P Lowe AD Rodríguez YY Murgia MV Dodd KA Rowland RR. Antigenic regions of African swine fever virus phosphoprotein P30. Transbound Emerg Dis. (2020) 67:1942–53. 10.1111/tbed.13533 32145150

[32] Gaudreault NN Madden DW Wilson WC Trujillo JD Richt JA. African Swine Fever Virus: An Emerging DNA Arbovirus. Front Vet Sci. (2020) 7:215. 10.3389/fvets.2020.00215 32478103 PMC7237725

[33] Chen X Chen X Liang Y Xu S Weng Z Gao Q. Interaction network of African swine fever virus structural protein p30 with host proteins. Front Microbiol. (2022) 13:971888. 10.3389/fmicb.2022.971888 36090090 PMC9451658

[34] Giménez-Lirola LG Mur L Rivera B Mogler M Sun Y Lizano S. Detection of African swine fever virus antibodies in serum and oral fluid specimens using a recombinant protein 30 (p30) dual matrix indirect ELISA. PLoS ONE. (2016) 11:e0161230. 10.1371/journal.pone.0161230 27611939 PMC5017782

